# Expansion of the osteocytic lacunar-canalicular system involved in pharmacological action of PTH revealed by AI-driven fluorescence morphometry in female rabbits

**DOI:** 10.1038/s41598-022-20793-5

**Published:** 2022-10-07

**Authors:** Aya Takakura, Takanori Sato, Ji-Won Lee, Kyoko Hirano, Ryoko Takao-Kawabata, Toshinori Ishizuya, Tadahiro Iimura

**Affiliations:** 1 Pharmaceuticals Research Center, Asahi Kasei Pharma Corporation, 632-1 Mifuku, Izunokuni, Shizuoka 410-2321 Japan; 2 Department of Pharmacology, Faculty and Graduate School of Dental Medicine, Hokkaido University, N13 W7, Sapporo, Hokkaido 060-8586 Japan

**Keywords:** Biological techniques, Cell biology, Diseases, Endocrinology

## Abstract

Osteoporosis is an age-related disorder that is characterized by reduced bone mass. Its prevention and treatment are important healthcare issues for maintaining social activity in aged societies. Although bone fractures mostly occur at sites of weakened cortical bone, pathophysiological and pharmacological evaluations of bone mass have tended to be predominantly assessed in trabecular bone. To statistically characterize cortical bone remodeling, we originally established multimode fluorescence imaging and artificial intelligence (AI)-driven morphometric analyses in six-month-old female rabbits with well-defined cortical remodeling, similar to that in humans. We evaluated three distinct administration frequencies of teriparatide [TPTD; human parathyroid hormone, hPTH (1–34)]: once (1/w), twice (2/w), and seven times (7/w) a week, with the same total dose (140 μg/kg/week). Our analyses revealed significant expansions of the osteocytic lacunar-canalicular system and Haversian canals accompanied by the development of cortical porosity and endosteal naïve bone formation induced by a frequent administration regimen (7/w) of TPTD; however, once-weekly (1/w) and twice-weekly (2/w) administration of TPTD showed little effect. These findings demonstrate a clear contrast between the effects of frequent and infrequent administration of TPTD on cortical bone metabolism and suggest that osteocytic bone remodeling is involved in the pharmacological action of PTH.

## Introduction

Osteoporosis is an age-related disorder characterized by reduced bone mass and impairment of bone quality, which increases the risk of bone fracture^1^. The dynamic equilibrium of bone formation and resorption maintains the bone mass and structure in healthy adult skeletons. Osteoporosis is an imbalanced condition of preponderance of bone-resorptive osteoclastic activity over bone-forming osteoblastic activity, caused by aging, loss of sexual hormones, inflammatory diseases, and disuse syndrome. Bone fractures, especially in the lumbar vertebrae and femoral neck, are highly associated with a reduction in quality of life and activities of daily living, and the prevention and treatment of osteoporosis is an important healthcare issue necessary to maintain social activity in aged societies.

Both in preclinical (animal models) and clinical studies, including pharmacological evaluation of anti-osteoporotic drugs, bone mass, and bone density have been classically assessed at common fracture bone sites such as the hip, wrist, and lumbar vertebrae. Growing recognition of the relevance of microarchitecture in bone deterioration and fragility has emerged, and its trend has been largely driven by innovative advance of imaging and related mechano-biological analyses^2–5^. Both trabecular and cortical bone are important determinants of bone strength, and it is currently known that thinner trabeculae and cortices are associated with the risk of vertebral and non-vertebral (appendicular bone) fractures, respectively^5–10^. Despite the fact that 80% of skeletal components are cortical bone and 80% of fractures occur at sites of weakened, thinned cortical bone^11^, assessment of trabecular bone has dominated the research, reducing the focus on the role of cortical bone in the pathophysiology of bone; this is often termed a “trabeculocentric” view of bone loss^6,10^. This biased view is possibly because trabecular bone is more morphometrically recognizable and assessable in preclinical (largely in mouse and rat models) and clinical studies, with changes in the microarchitecture of the cortices being under resolution. This is also related to the fact that the cortical bone in larger vertebrates, including humans, exhibits obvious biological turnover (cortical bone remodeling), while smaller species such as mice and rats, commonly used model animals in bone studies, show little cortical turnover with few typical structures of osteons throughout their lives. Therefore, there is an increasing need for innovative assessments of cortical architecture with high-resolution imaging-based analyses using larger remodeling animal models to move beyond the classical method.

The remodeling of cortical bone largely takes place on bone surfaces surrounding vascular pores called Haversian canals, which encase neurovascular bundles, with continuous removal and replacement of a small amount of existing bone^12–15^. The small fractions of cells responsible for an individual remodeling event are called basic multicellular units (BMUs), which physiologically couple and balance bone resorption and formation by osteoclasts and osteoblasts, respectively, aiding with mineral exchange and maintaining mechanical integrity^16^. Imbalance in cortical remodeling, with bone resorption outweighing bone formation, results in pathological expansion of the scale of Haversian canals, which can be the main cause of an increase in cortical porosity observed in osteoporotic bone^4,17^. Therefore, it is important to assess the spatiotemporal regulation of BMUs in the pathophysiology of cortical bone.

Cortical bone remodeling possibly involves bone turnover regulated by osteocytes, the most abundant bone cells in the adult skeleton^18^. Osteocytes form an osteocyte network in the mineralized bone matrix, with their dendrites enabling them to connect to each other, blood vessels, and bone surface cells (osteoclasts and osteoblasts, including lining cells), thus establishing global cellular communication in bone tissue. Osteocytes and their dendrites are encased by micro-bone cavities and canals called osteocytic lacunae and canaliculi, respectively. The surface of the osteocytic lacunar-canalicular network is estimated to be several times larger than the bone surface^19^. Current findings have elucidated the molecular mechanisms involved in the bone resorptive function of osteocytes: they participate in “osteocytic osteolysis”, recognized by enlarged osteocytic lacunae^18^. Although osteocytic osteolysis is well documented in lactation and hyperparathyroidism, the involvement of osteocytic osteolysis in the pathophysiology of bone loss and the pharmacological action of anti-osteoporotic drugs remains largely unknown. This necessitates the development of methods to statistically assess this biological phenomenon.

Teriparatide [TPTD; human parathyroid hormone, hPTH (1–34)] is clinically used for the treatment of osteoporosis because of its anabolic effects on bone when administered intermittently. The pharmacological effects of TPTD fundamentally ensure bone turnover by stimulating both bone formation and resorption. In addition, intermittent administration of TPTD increases net bone mass by enhancing bone formation, which is dominant over bone resorption, while continuous TPTD administration reduces bone mass with stimulating bone resorption over bone formation. We recently reported that intermittent once-weekly TPTD administration in ovariectomized (OVX) cynomolgus monkeys enhanced bone quality by inducing synchronized bone formation and a linear arrangement of collagen fibers in vertebral trabeculae^20^.

Three distinct regimens of TPTD administration (once-daily, once-weekly, and twice-weekly) are currently available for the treatment of osteoporosis. The once-weekly regimen was developed with less stimulation of bone resorption, compared to the effect of the once-daily regimen that leads to marked increases in the markers of both bone formation and resorption^21–24^. Patients receiving the once-weekly regimen must either be outpatients or inpatients, while the once-daily regimen can be self-administered by the patient as a home recuperation treatment^25,26^. Therefore, the twice-weekly regimen with a self-injection device that was recently approved in Japan is expected to reduce the burden on patients from both hospital visits and frequent self-injection^22^. These three distinct regimens were selected based on the requirements and clinical conditions of each patient.

The effects of the approved once-daily and once-weekly regimens on the reduction of the risk of vertebral fractures appear to be comparable. However, accumulated studies have demonstrated that distinct administration frequencies, as well as dosing of TPTD, affected the microarchitecture and strength of bone in clinical^27^ and animal^28–32^ models. Our previous study in an OVX rat model reported that a high frequency of administration of TPTD (6 μg/kg three times daily) significantly developed cortical porosity in vertebral bones associated with increased bone resorption, whereas lower frequency administrations (such as 30 μg/kg once-daily and 30 μg/kg three times weekly) did not^28^. Another study in rabbit femurs with a well-developed Haversian system in their cortices observed that frequent administration of TPTD (40 μg/kg once-daily) induced a significantly higher score of cortical porosity than low-frequency administration (280 μg/kg once-weekly), even though the total weekly doses of these two regimens were equivalent^29^.

The primary objective of our study was to statistically characterize cortical porosity, while focusing on Haversian and osteocytic bone remodeling, by providing multimode fluorescence imaging and artificial intelligence (AI)-driven morphometric analyses in rabbits (the smallest commonly employed laboratory animal with well-defined cortical remodeling similar to humans) using three distinct dosing frequencies of TPTD (once, twice, and seven times a week) with the same total dose (140 μg/kg/week).

## Results

**Figure 1:**
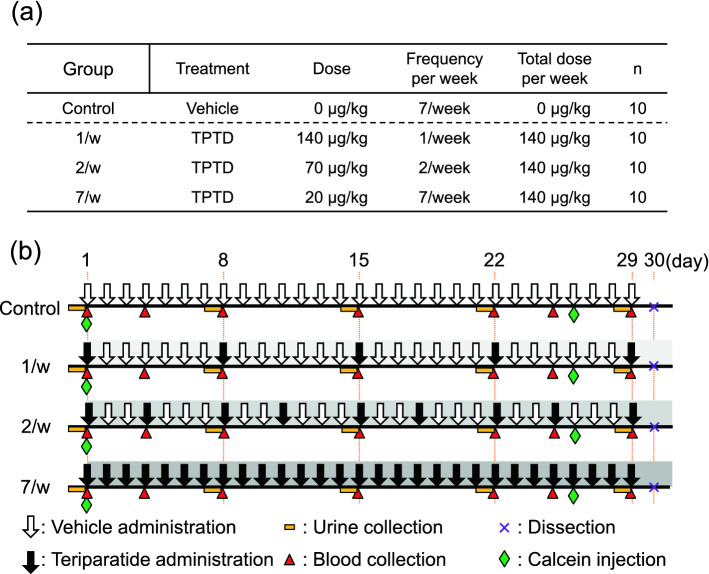
The time course and regimen settings of Teriparatide (TPTD) administration and samplings. (**a**) Six-month-old female rabbits were divided into four groups: (1) Control group (vehicle administration) group, (2) 1/w group (once-weekly administration of 140 μg/kg of TPTD), (3) 2/w group (twice-weekly administration of 70 μg/kg of TPTD), (4) 7/w group (once-daily administration of 20 μg/kg of TPTD). (**b**) The administration schedules of TPTD (black arrows) or vehicle (white arrows) are demonstrated in each regimen setting. Timings of urine and blood collections, bone dissection, and bone labeling with calcein are also indicated.

**Figure 2:**
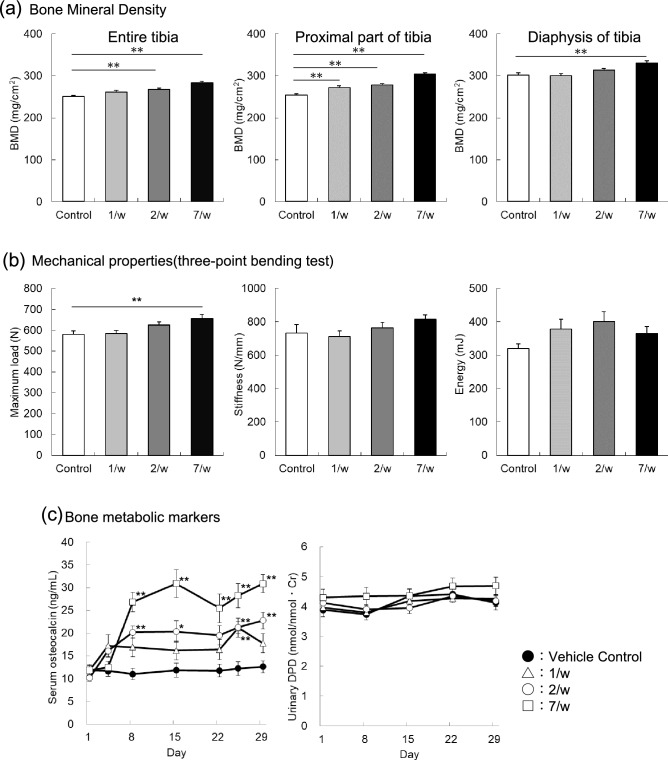
The bone mineral densities (BMDs) and mechanical properties of tibiae and changes in the bone metabolic markers in serum and urine. (**a**) The BMDs of the total, proximal, and diaphyseal portions of the tibiae measured by dual-energy X-ray absorptiometry are statistically compared. (**b**) Mechanical parameters such as, maximum load (N), stiffness (N/mm), and energy (mJ) are statistically compared. (**c**) Levels of serum osteocalcin and urine deoxypyridinoline (DPD) are plotted against the time course of TPTD administration. Data (a-c) are shown as mean + SE. * Indicates P < 0.05 and ** indicates P < 0.01 vs. control (Dunnett’s test).

In this study, three pharmacological regimens of TPTD [140 μg/kg (1/w), 70 μg/kg (2/w), and 20 μg/kg (7/w)] were administered to six-month-old female rabbits, with equivalent total weekly doses (Fig. 1). Doses given to the rabbits were adjusted for higher metabolic rate of small experimental animals than that in humans, with considering toxicity as previously described^29^. We first measured the bone mineral densities (BMD) of the tibial bones using dual-energy X-ray absorptiometry (DXA) (Fig. 2a). Scores of the total, proximal, and diaphyseal portions of the tibiae showed gradual increases in an administration frequency-dependent manner. All measured BMD scores and maximum load in the bending test of the 7/w group showed a significant increase (Fig. 2a,b). The mechanical strength scores for stiffness and energy did not show significant differences among the experimental groups. The time course changes in the serum level of osteocalcin (OC) showed a clear increase three days after the initial administration, while the urinary levels of deoxypyridinoline (DPD) in all experimental groups were sustained at similar levels over the time course observed (Fig. 2c).

**Figure 3:**
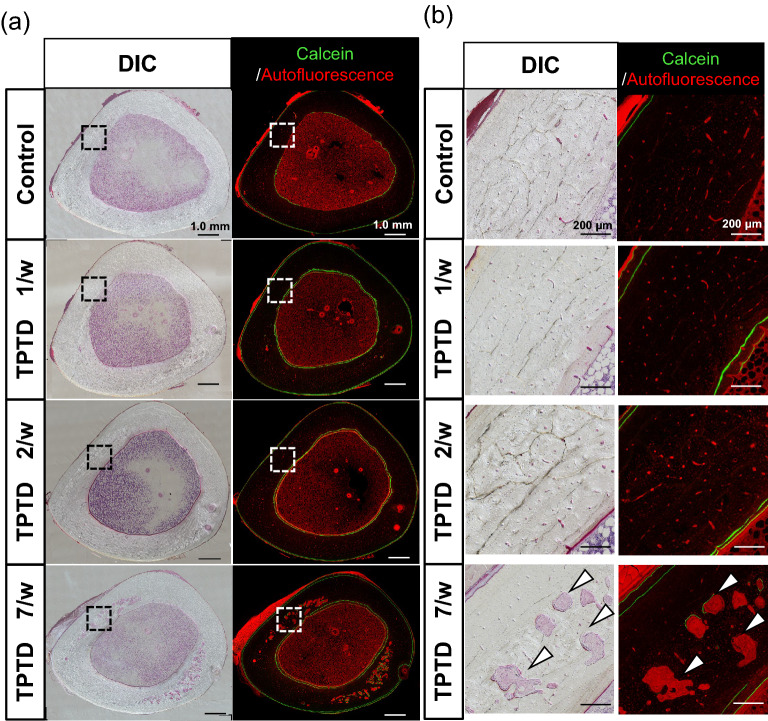
Histological observation of the formation of cortical bone porosity by fluorescence and differential interference contrast (DIC) images of rabbit tibia. (**a**) Representative whole fluorescence (right panels) and corresponding DIC images (left panels) of tibial bone sections obtained from the control, 1/w, 2/w, and 7/w groups are shown. Scale bars indicate 1.0 mm. (**b**) Magnified images framed by dotted boxes in the images shown in (**a**) are shown. White arrowheads indicate typical portions of cortical bone porosity. Scale bars indicate 200 µm.

**Figure 4:**
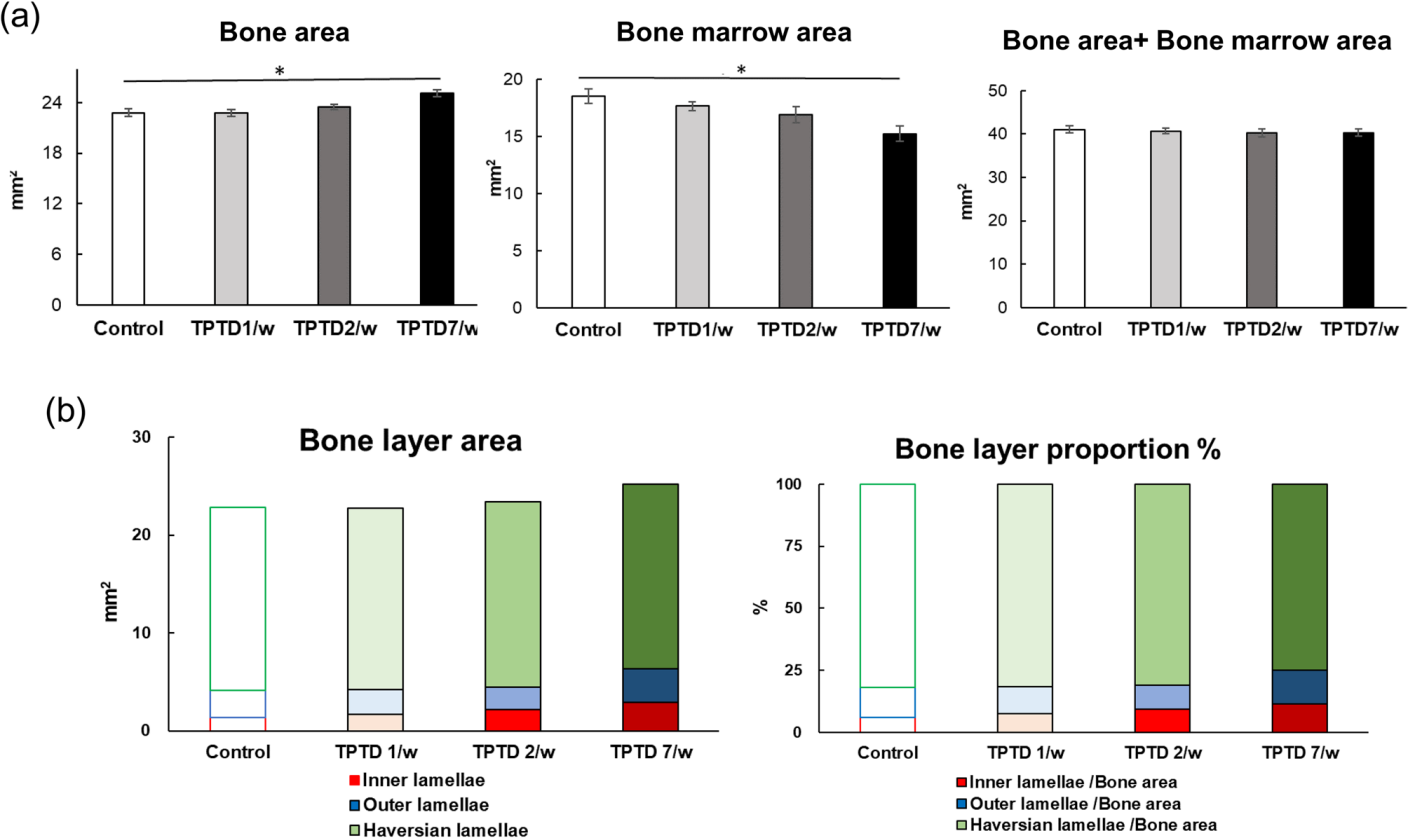
Bone anabolic effect of TPTD on rabbit tibial bone layers. (**a**) Scores of bone area, bone marrow area, and bone area with bone marrow area are statistically compared. Data are shown as mean ± SE. *Indicates P < 0.05 vs. control (Dunnett’s test). (**b**) Average areas and relative area portions of inner, outer, and Haversian lamella of tibial bone sections obtained from the four regimen groups are compared.

**Figure 5:**
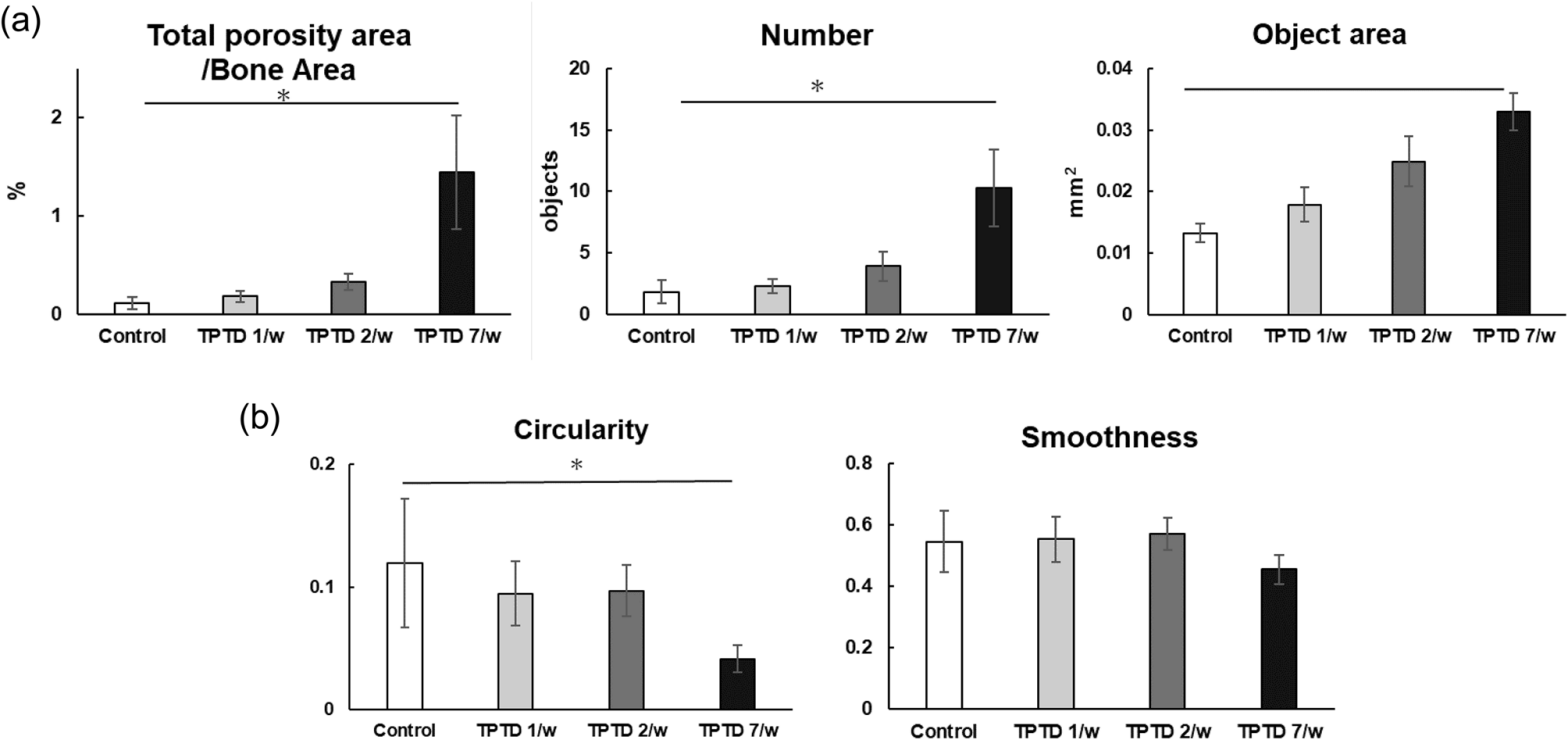
The quantitative topological analysis of cortical bone porosity in rabbit tibia. Topological parameters of bone porosity, such as (**a**) total porosity area/bone area, number, object area, circularity, and (**b**) smoothness, were measured and statistically compared. Data are shown as mean ± SE. *Indicates P < 0.05 vs. control (Dunnett’s test).

Full-view observation of transverse tibial bone sections using the bright field of differential interference contrast (DIC) and fluorescence imaging demonstrated obvious development of cortical porosity, especially in the specimens obtained from the 7/w group (arrowheads in Fig. 3b). Therefore, we took advantage of the semiautomatic morphometric recognition system that we previously developed (Sato 2021) to conduct histological measurements of the areas of bone porosity (Figs. 4 and 5, Supplementary Fig. S1). Scores of bone area and bone marrow area of 7/w mice showed significant increases and decreases, respectively, compared to those of other groups, suggesting obvious inward bone formation (Fig. 4a). Consistently, tissue layer measurements of the inner, outer, and Haversian lamellae showed thicker inner lamellae in the 7/w group compared to those in the other groups (Fig. 4b). These observations were also consistent with micro-CT-based parameters of tibial mid-shafts, such as the cortical bone ratio (Cv/Av, %), cortical bone thickness (Ct.Th, μm), external length (Ex.L, μm), and internal length (In.L, μm) (Supplementary Fig. S2). The proportion of porosity area to bone area and the number and average size of pores in the 7/w group showed significantly higher scores than those in the other groups (Fig. 5a). A micro-CT-based parameter of cortical porosity (Ct.P, %) showed a similar tendency, while the moment of inertia (moment, mm^5^) showed a slight decrease in the 7/w group compared to those in the 1/w and 2/w groups (Supplementary Fig. S2). The morphometric parameters of circularity and smoothness demonstrated non-circular and rough margins of porosity developed in the 7/w group, suggesting active bone resorption. (Fig. 5b).

**Figure 6:**
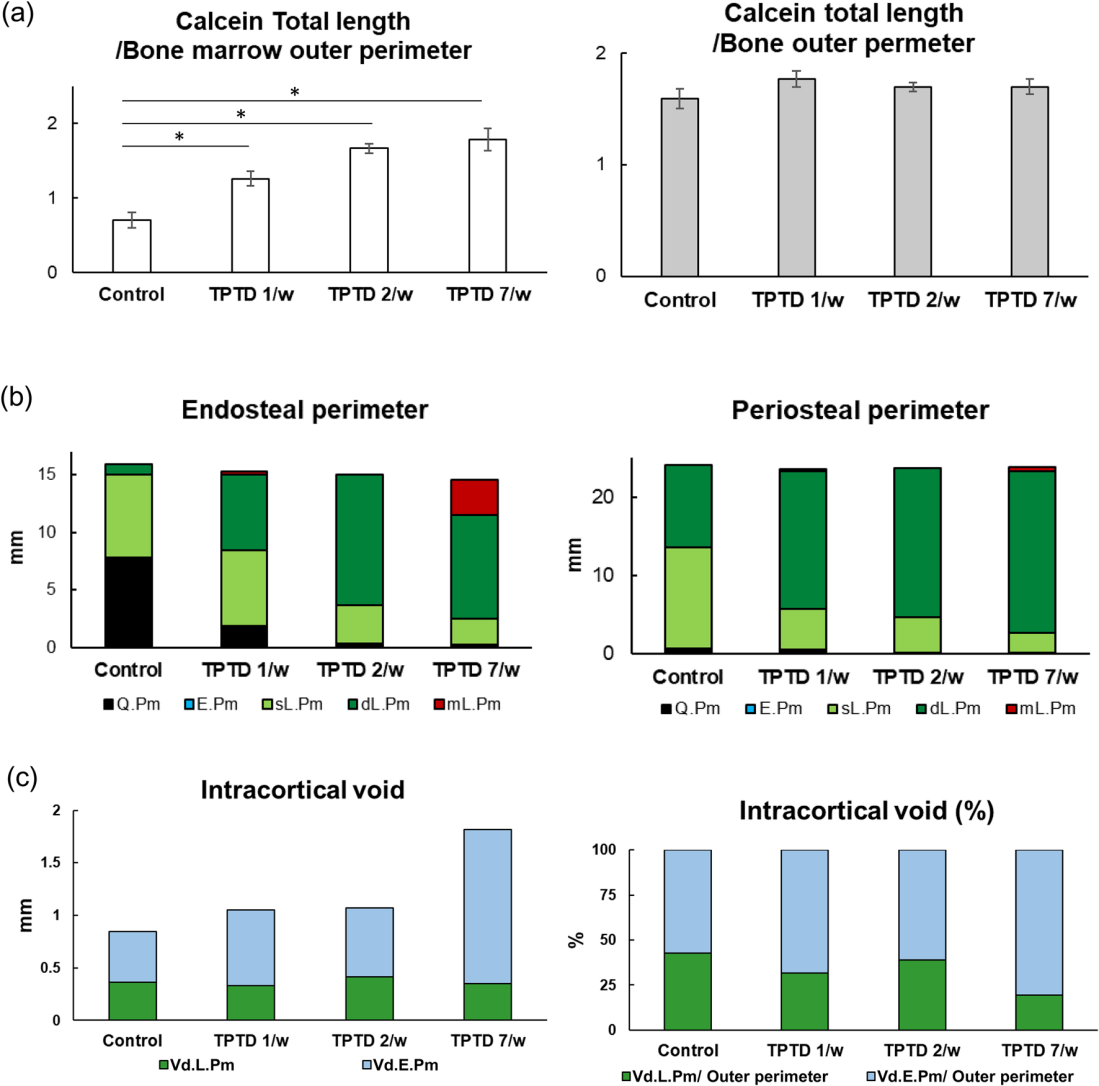
The histomorphometric analysis of the cortical bone parameters. (**a**) The topological parameters of calcein signals, such as calcein total length/bone marrow outer perimeter, and bone outer perimeter, are measured and statistically compared. Data are shown as mean ± SE. *Indicates P < 0.05 vs. control (Dunnett’s test). (**b**) The endosteal perimeter, periosteal perimeter, and (**c**) intracortical void are compared. *mL.Pm* multiple-labeled perimeter, *dL.Pm* double-labeled perimeter, *sL.Pm* single-labeled perimeter, *E.Pm* eroded perimeter, *Q.Pm* quiescent perimeter, *Vd.L.Pm* void-labeled perimeter, *Vd.E.Pm* void-eroded perimeter.

Next, we applied the semiautomatic morphometric recognition system to analyze calcein-labeling patterns of the established outer and inner bone surfaces and porosity surfaces (Fig. 6, Supplementary Fig. S1)^20^. The ratio of inner bone-forming surface marked by labeled calcein length to bone marrow outer perimeter showed a significant increase in an administration frequency-dependent manner, while the outer bone-forming surface did not show significant change (Fig. 6a). This was consistent with our observations by histomorphometry and micro-CT (Figs. 3 and 4, Supplementary Fig. S2), suggesting inward bone formation by TPTD administration. Accordingly, the double-labeled surface of the inner (endosteal) perimeter in the three groups increased compared to that in the control (Fig. 6b). Notably, a multiple-labeled surface was specifically observed in the daily regimen of 7/w (marked in red in Fig. 6b, arrowheads in Supplementary Fig. S3). The double-labeled surface of the outer perimeter in the three groups also increased. The eroded perimeter of intracortical voids in 7/w was markedly increased (Fig. 6c), whereas the ratio of labeled to eroded perimeters of intracortical voids appeared to be comparable in all groups.

**Figure 7:**
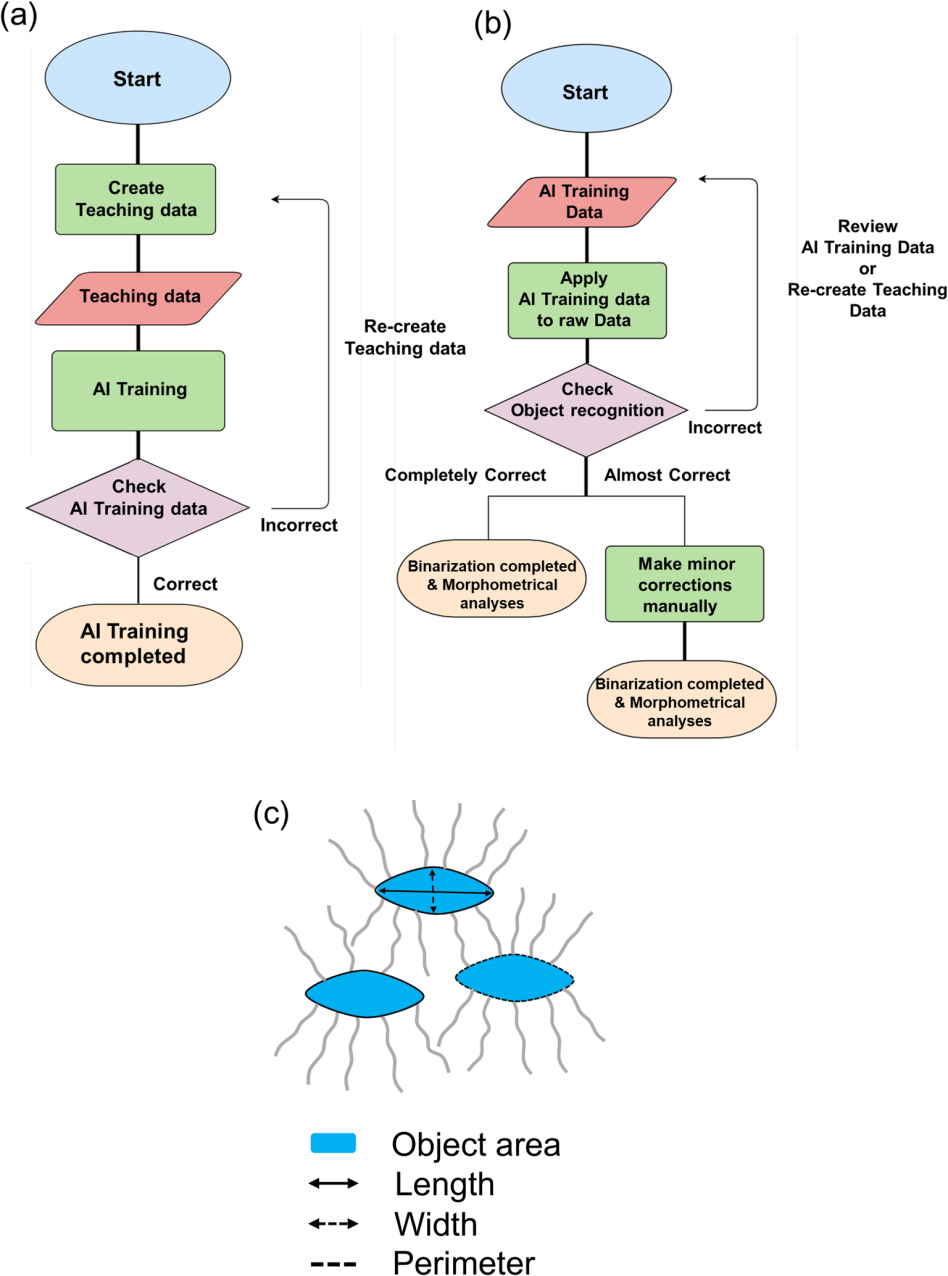
The methodological flow charts of artificial intelligence (AI)-driven two-dimensional morphometrical analyses (**a**,**b**). Flow charts of (**a**) AI training and (**b**) AI-driven object recognition are shown. (**c**) The parameter setting of two-dimensional morphometric analyses of osteocytic lacunae are demonstrated. Parameters, such as object area, length, width, and perimeter of osteocytic lacunae are calculated.

**Figure 8:**
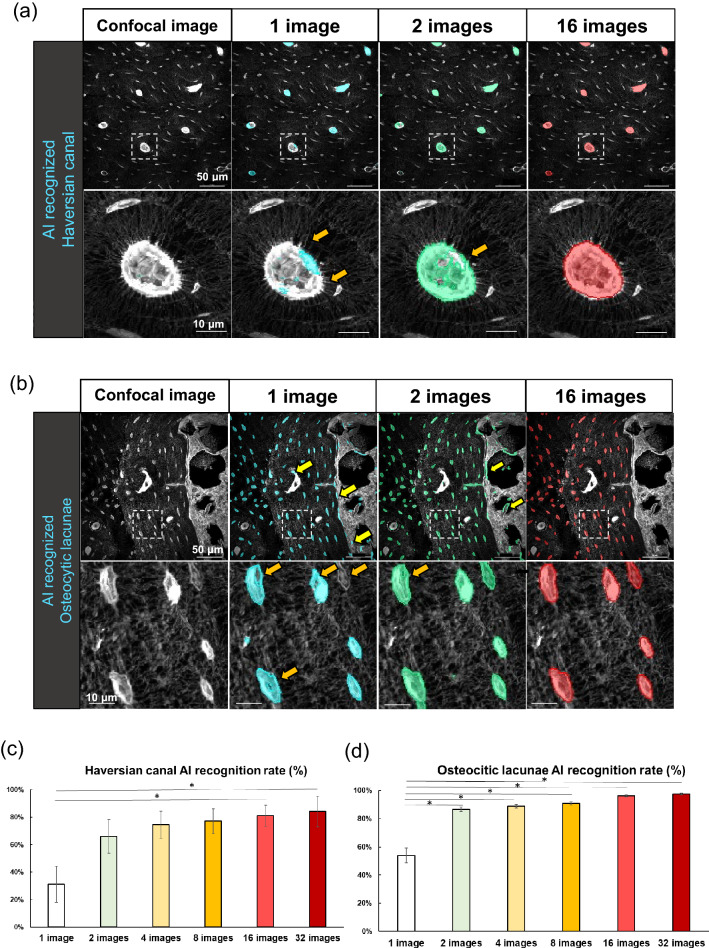
Evaluation of accuracy in AI-driven morphological recognition with distinct numbers of training images. (**a**,**b**) The representative images of AI-driven morphological recognition in Haversian canal (**a**) and osteocytic lacunae (**b**) are shown. Low magnification views (upper panels) and high magnification images (lower panels) framed by white dotted boxes in upper panels are shown. Scale bars in upper and lower panels indicate 50 µm and 10 µm, respectively. The results of AI-driven segmentations with 1, 2, and 16 training images are indicated in distinct colors of blue, green, and red, respectively. Yellow and orange arrows indicate extra morphological extraction and insufficient recognition of objects, respectively. (**c**,**d**) The successful ratios of AI-driven recognition with distinct numbers of training images of Haversian canal (**c**) and osteocytic lacunae (**d**) are scored and statistically compared. Data are shown as mean ± SE. *Indicates P < 0.05 vs. 1 image (Dunnett’s test).

**Figure 9:**
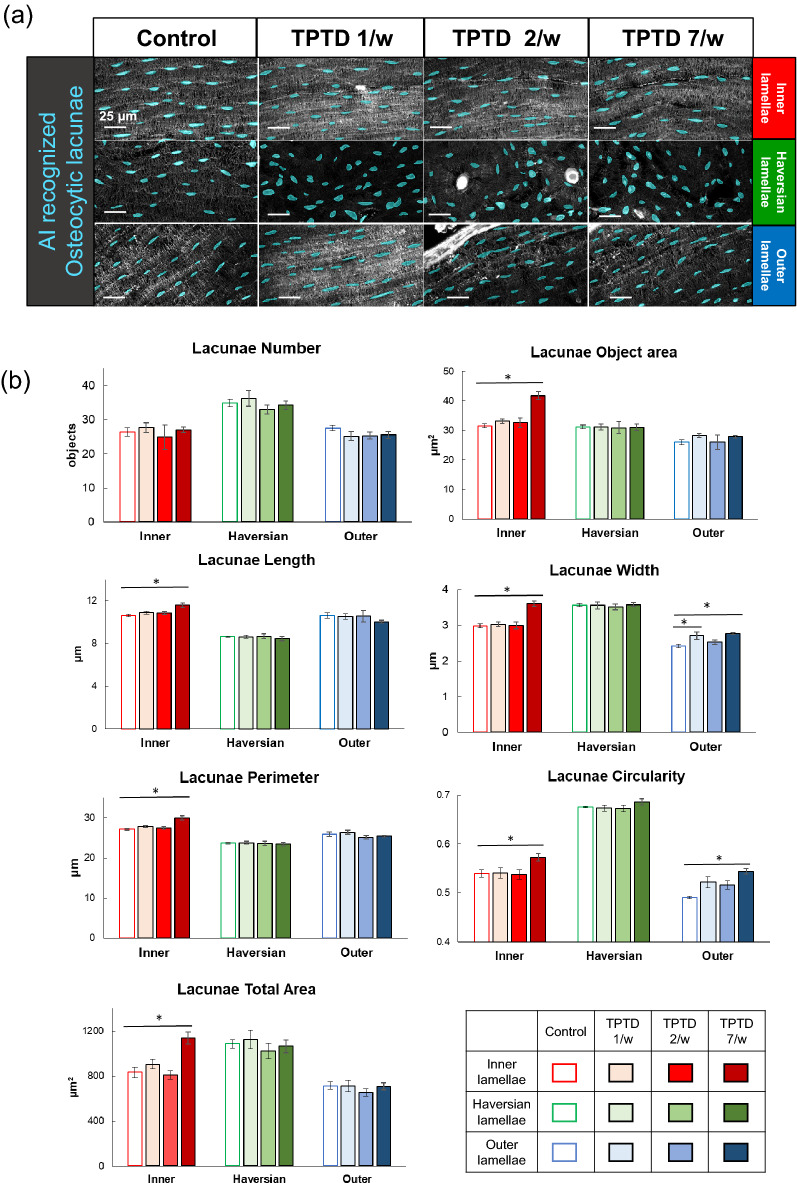
Quantitative two-dimensional analysis of Haversian canals. (**a**) The representative images of AI-recognized Haversian canals obtained from four regimen setting groups are shown. Scale bars indicate 50 µm. (**b**) Topological parameters of Haversian canal, such as number, object area, length, width, outer perimeter, total area, and circularity are measured and statistically compared. Bars are color-coded as indicated in the bottom-right table.Data are shown as mean ± SE. *Indicates P < 0.05 vs. control (Dunnett’s test).

**Figure 10:**
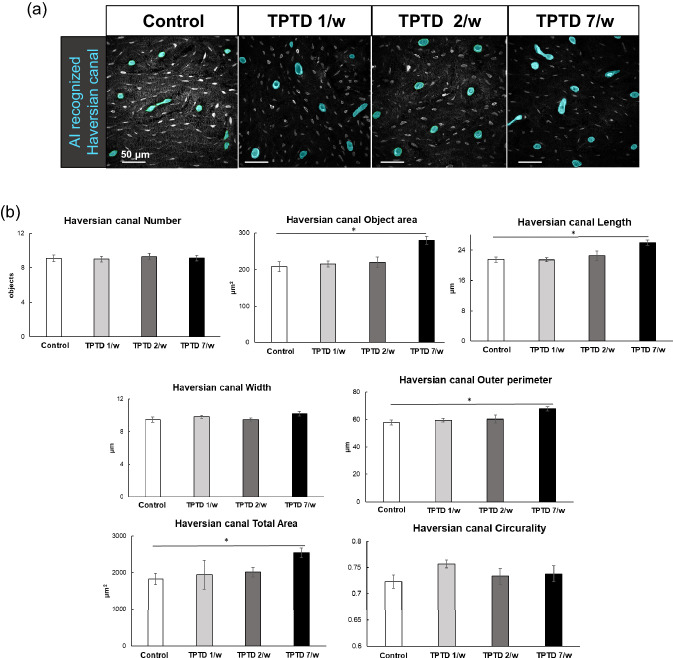
Quantitative two-dimensional analysis of osteocytic lacunae. (**a**) Representative images of AI-recognized osteocytic lacunae in the three distinct tibial bone layers obtained from four regimen setting groups are shown. Scale bars indicate 25 µm. (**b**) Topological parameters of osteocytic lacunae, such as number, object area, length, width, outer perimeter, total area, and circularity are measured and statistically compared. Data are shown as mean ± SE. *Indicates P < 0.05 vs. control (Dunnett’s test).

To test the possible involvement of osteocytic osteolysis and bone remodeling of Haversian canals in the pharmacological action of TPTD, we established an AI-driven two-dimensional morphometric analysis of Villanueva-stained bone sections (Figs. 7, 8, 9, 10, Supplementary Fig. S4), in which autofluorescence signals in mineralized bone tissue demarcated the osteocytic lacunar-canalicular system and Haversian canals, as described previously^20,28^. First, we provided manually binarized teaching data on osteocytic lacunae and Haversian canals for machine learning (Fig. 7). Next, we evaluated the success ratio of AI-driven automatic morphometric recognition using distinct amounts of teaching data (Fig. 8a,b). The success ratio increased in a number-dependent manner in the teaching data, and 16 or more teaching data showed significantly satisfying morphometric recognition for osteocytic lacunae and Haversian canals (Fig. 8c,d). Osteocytic lacunae in three layers (inner, outer, and Haversian lamellae) of the cortical bone were automatically recognized after machine training. The shape and spatial arrangement of the lacunae in each lamella showed a distinct pattern (Fig. 9a). Therefore, we compared morphometric parameters, such as number, area, length, width, perimeter, circularity, and total area, in the three distinct lamellae (Fig. 9b). All parameters, except the number of lacunae in the 7/w group, showed significantly higher scores than those in the other regimens, which were characteristically observed in the inner lamella. The lacuna width and circularity in the outer lamella tended to be higher after TPTD administration. Comparisons of these parameters among the three cortical layers with control and distinct TPTD administration frequencies also demonstrated similar patterns but a marked increase in the size and other related parameters of the lacunae in the 7/w group, specifically in the inner lamellae (Supplementary Fig. S4). These morphometric analyses demonstrated bone site-specific expansion of osteocytic lacunae in the most frequent administration setting (7/w) of TPTD.

We also applied AI-driven two-dimensional morphometric analyses to the Haversian canals (Fig. 10a). The Haversian canal object area, length, perimeter, and total area were significantly enlarged in specimens of 7/w, compared to those in the vehicle control, 1/w, and 2/w, while the number and width of the Haversian canals showed significant changes among the groups (Fig. 10b).

**Figure 11:**
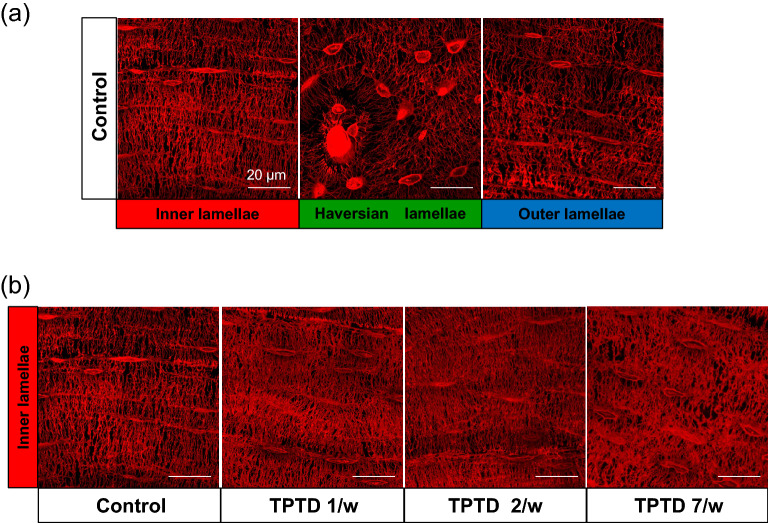
The representative images of three-dimensional confocal images of osteocytic lacunae. (**a**) Representative images of osteocytic lacunae in the distinct tissue layers of inner, outer, and Haversian lamella in the control group are shown. (**b**) The representative images of osteocytic lacunae in the layer of inner lamellae in the distinct regimen settings of TPTD administration are shown. Scale bars in (**a**,**b**) indicate 20 µm.

**Figure 12:**
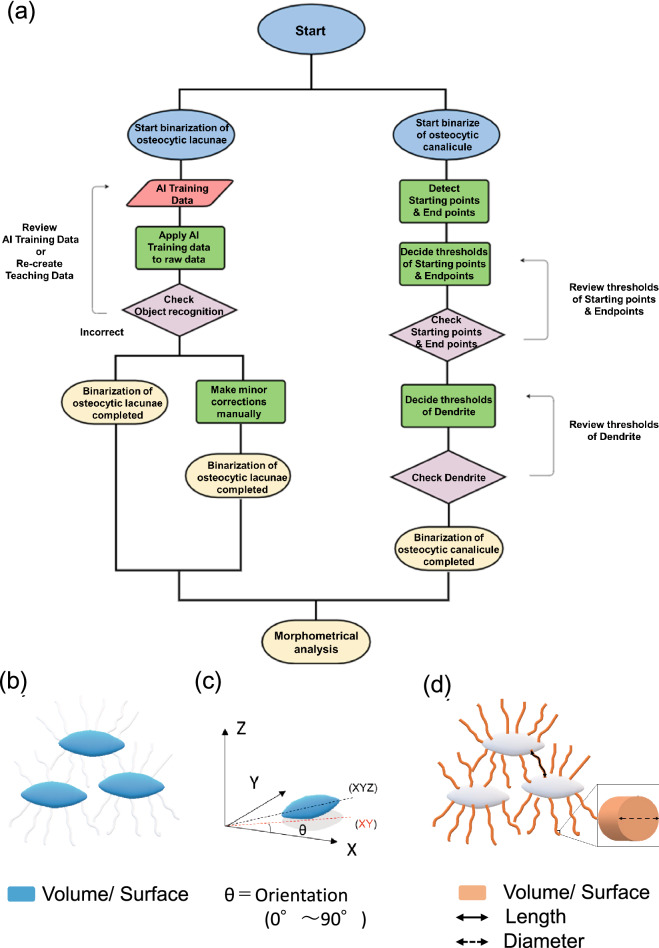
The methodological flow charts of artificial AI-driven three-dimensional morphometrical analysis of the osteocytic lacunar-canalicular system. (**a**) Flowchart of AI-driven three-dimensional recognition of osteocytic lacunae and canaliculi. The osteocytic lacunae and canaliculi are three-dimensionally binarized by the AI-driven recognition system and the three-dimensional osteocytic canaliculi were binarized by three-dimensional tracing. (**b**–**d**) The parameter setting of three-dimensional morphometric analyses of osteocytic lacunae (**b**,**c**) and canaliculi (**d**) are graphically demonstrated. (**b**,**c**) Parameters such as volume, surface, and orientation of osteocytic lacunae are measured. (**d**) Parameters such as volume, surface, length, and diameter of osteocytic canaliculi are measured.

**Figure 13:**
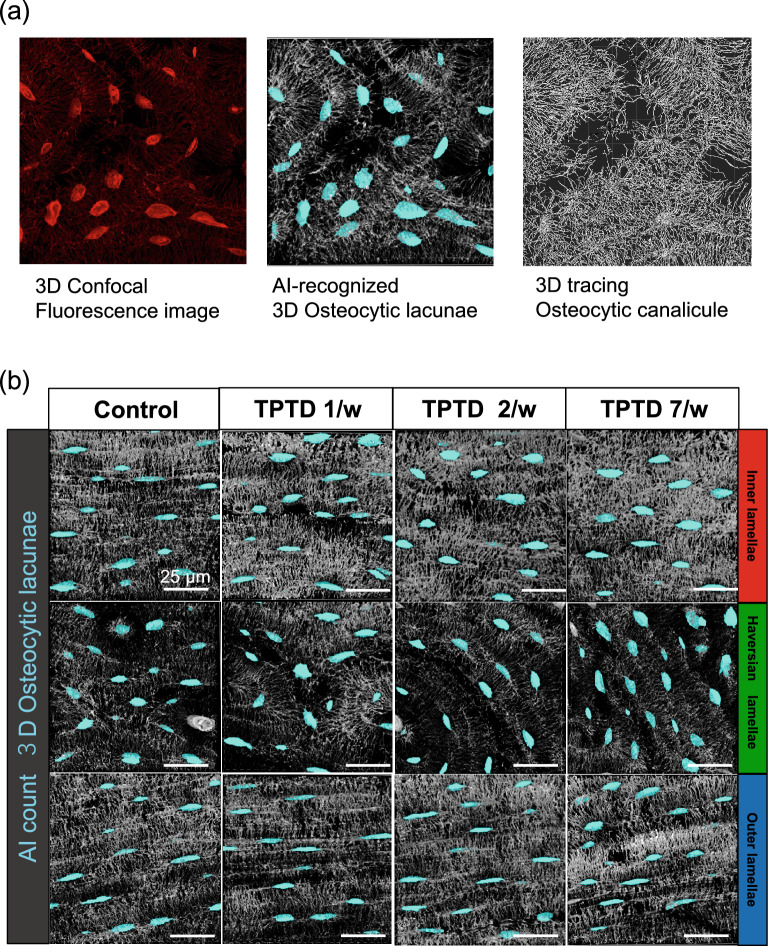
The representative images of the three-dimensionally AI-recognized osteocytic lacunar-canalicular system. (**a**) Representative images of fluorescence and corresponding AI-recognized osteocytic lacunae and tracing of osteocytic canaliculi are shown. (**b**) Representative images of three-dimensionally AI-recognized osteocytic lacunae in the distinct tissue layers of inner, outer, and Haversian lamella in the four regimen settings of TPTD administration are shown. Scale bars indicate 25 µm.

**Figure 14:**
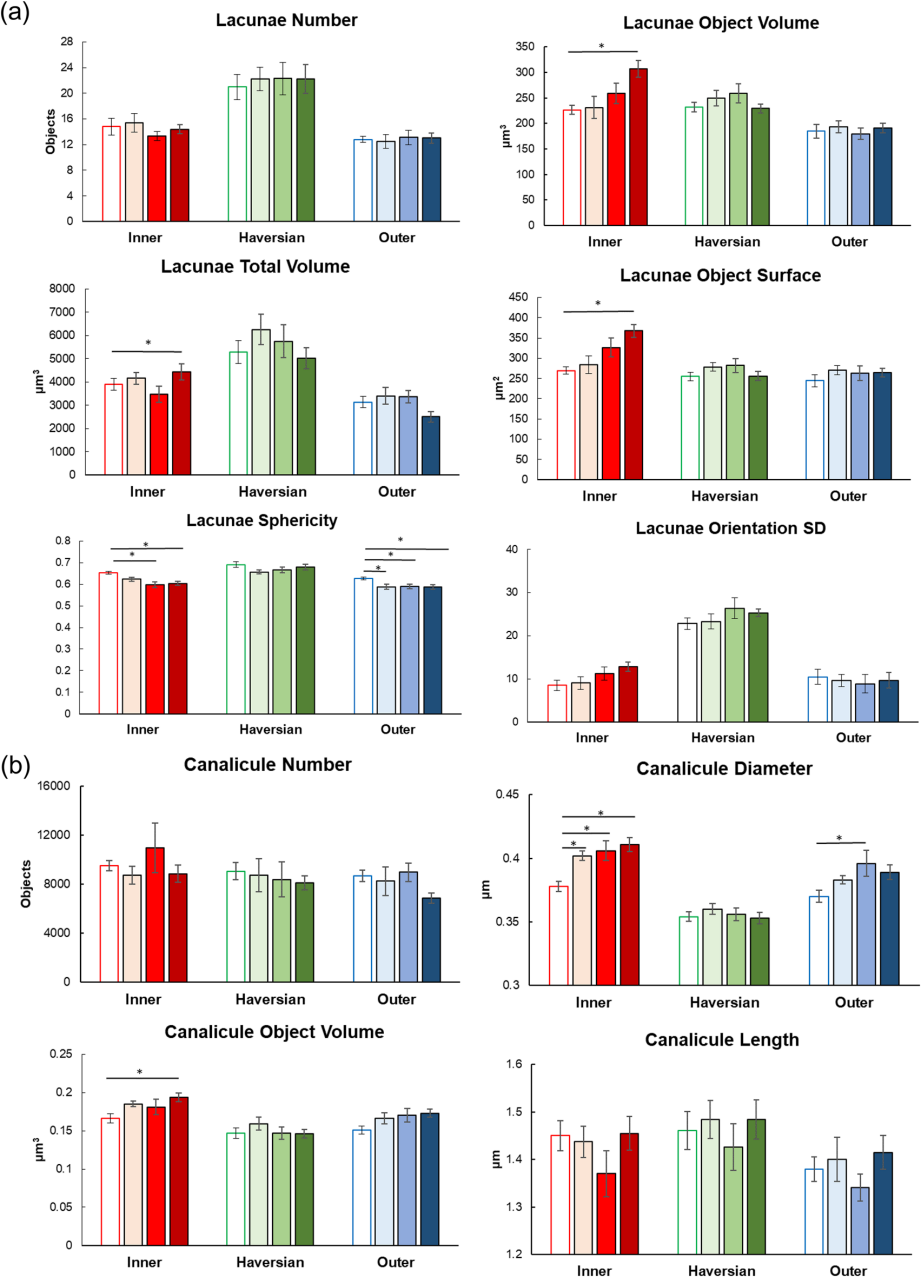
Quantitative three-dimensional analysis of the osteocytic lacunar-canalicular system. (**a**) Topological parameters of osteocytic lacunae, such as number, object volume, total volume, object surface, sphericity, and orientation SD (standard deviation) are measured and statistically compared. Data are shown as mean ± SE. * Indicates P < 0.05 vs. control (Dunnett’s test). (**b**) Topological parameters of osteocytic canaliculi, such as number, diameter, object volume, and length are measured and statistically compared. Bars are color-coded as shown in Fig. 9. Data are shown as mean ± SE. *Indicates P < 0.05 vs. control (Dunnett’s test).

We further applied the AI-driven morphometric analyses to three-dimensional high-resolution imaging and successfully separated osteocytic canaliculi from lacunae (Figs. 11, 12, 13, 14, Supplementary Figs. S5, S6). Consistent with the two-dimensional analyses, parameters such as object volume, surface, and total volume of lacunae were significantly augmented by 7/w TPTD administration, specifically in the inner lamella, while the number and orientation of lacunae did not show significant differences (Figs. 13, 14a). As were observed in our two-dimensional analyses, comparisons of these parameters among the three cortical layers also demonstrated similar tendencies in control and distinct TPTD administration frequencies except a significant increase in the volume and surface of the lacunae in the 7/w group, specifically in the inner lamellae (Supplementary Fig. S5). We further established and compared the morphometric parameters of osteocytic canaliculi (Fig. 14b). Canalicular diameter and volume were significantly increased by 7/w TPTD administration in the inner lamella, whereas the number and length of lacunae in this bone site did not show significant changes. Comparisons of these parameters among the three cortical layers demonstrated fundamentally similar patterns in control and distinct TPTD administration frequencies (Supplementary Fig. S6). Canalicular diameter and volume in the outer lamella tended to increase with TPTD administration, which was consistent with the two-dimensional analyses. These three-dimensional morphometric analyses demonstrated bone site-specific expansion of osteocytic lacunae and canaliculi after 7/w TPTD administration.

## Discussion

We designed this study to determine whether different frequencies of TPTD administration affected the microstructure of cortical bone, such as cortical porosity, bone marrow fibrosis, and the osteocytic lacunar-canalicular system, by observing female rabbit tibiae.

We monitored the time-course changes in serum OC levels after administration and urinary DPD levels on days 1 to 29 after the initial administration of TPTD. OC levels were augmented by any of the TPTD administration regimens compared to those in vehicle administration, suggesting stimulated bone formation by TPTD. Notably, the daily TPTD administration (7/w) maintained higher levels of OC over 25 μg/mL throughout the administration period after day 8, whereas the twice- and once-weekly administrations (2/w and 1/w, respectively) induced comparable levels of serum OC at 15–20 μg/mL. The urinary DPD levels in the twice- and once-weekly administrations (2/w and 1/w, respectively) were comparable to those in the vehicle control, while that in daily TPTD administration (7/w) was slightly (albeit not significantly) higher than those throughout the monitored period. Similarly, differences in bone metabolic markers between distinct administration frequencies have also been shown in previous clinical studies^33–36^. These changes in bone metabolism markers are possibly associated with the development of cortical porosity, marrow fibrosis, and expansion of the osteocytic lacunar-canalicular system, which will be discussed in the following sections.

We observed a significant expansion of the Haversian canals, especially in specimens from the group administered daily TPTD (7/w). This was specifically observed in the Haversian layer, in a clustered manner, but not in the other cortical layers of the outer and inner lamellae. Harrison et al. described the non-random appearance of cortical pores in rabbit tibiae following daily administration (30 μg/kg) of hPTH (1–34) for 4 weeks by three-dimensional micro-CT analysis^4^. Cortical pores were observed in the mid-cortex and were frequently clustered, which is very similar to the morphometric observations of the current study. The adult mid-cortex was described to contain remnants of developmental tissues evidenced by inlands of calcified cartilage^37,38^, and its unique Haversian structure suggests tight functional coordination with the vascular system and bone metabolism throughout life. The Haversian canal expansion and the mid-cortical pores induced by daily administration of the PTH contrast with previous reports by Zebaze et al. and Hirano et al., both of whom observed increased porosity primarily adjacent to the marrow cavity in the proximal femur and the mid shaft of the tibia, respectively, in rabbits^27,31^. These endosteal pores reported by them could be related to endosteal naïve bone formation and the expanded lacunar-canalicular system, which will be discussed in the subsequent sections.

A significant expansion of the osteocytic lacunar-canalicular system was observed in the cortical layer of the inner lamellae of specimens, upon daily TPTD administration (7/w). This is the first qualitative evidence of the possible involvement of osteocytic osteolysis in the pharmacological actions of daily TPTD administration. Evidence of osteocytic osteolysis in hyperparathyroidism has been provided by histological evaluation of osteocytic lacunae in iliac crest biopsies^39,40^. The notion that PTH mobilizes calcium by removing pericanalicular minerals was provided in the 1970s by studies regarding PTH administration to thyroparathyroidectomized rats^41,42^. Recent findings using contact microradiography and synchrotron X-ray tomography confirmed pericanalicular demineralization in rodents continuously treated with PTH^43,44^. Although it is well accepted that PTH target cells are osteoblastic lineage cells, the precise stage of differentiation of the target cells has not been well characterized^45^. Nevertheless, genetic activation or deletion of the PTH receptor under the control of DMP1 promoters specifically activated in late osteoblasts and early osteocytes in mice demonstrated the functional relevance of PTH receptors in osteocytes as regulators of osteoclastogenesis through their regulatory expression of RANKL and OPG^46,47^. These findings suggest that osteocytes are pharmacological targets of TPTD; however, it remains unclear how continuous or frequent administration, such as daily administration of TPTD, mobilizes calcium from the perilacunar-canalicular matrix.

Endosteal bone formation induced by PTH administration has been well documented in animal^4,29–31,48,49^ and human^50,51^ studies. Our research group previously reported that weekly administration of TPTD with the same (140 μg/kg/week) or double (280 μg/kg/week) dose formed new lamellar bone that appeared to be well harmonized with the previously existing inner lamellae, while daily administration (20 μg/kg/day, 40 μg/kg/day) induced trabecular-like woven bone on the endosteal surface^29^. This study demonstrated that the twice-weekly administration (70 μ/kg) of TPTD induced endosteal lamellar bone similar to that induced by daily administration. Harrison et al. described trabecularized bone formation on the endosteal surface of rabbit tibiae induced by the daily administration (30 μg/kg) of human PTH (1–34). These findings suggest that continuous or frequent administration, such as daily administration of TPTD, induces bone turnover/growth on the endosteal surface. We often observed that this trabecular-like, poorly developed bone contained pores, some of which were fused with vascular pores or even with expanded canaliculi. It is possible that cortical pores in the endosteal portion described in previous studies were formed primarily due to poorly developed endosteal bone, which shows clear contrast with the mid-cortical pores by the expansion of Haversian canals.

In this study, we conducted multimode fluorescence imaging and AI-driven morphometric analyses, providing a new way to evaluate cortical bones. We observed an inner cortical layer-specific expansion of the osteocytic lacunar-canalicular system, induced by frequent administration (7/w) of TPTD, suggesting osteocytic bone remodeling in the pharmacological action of PTH, which likely contributes to cortical porosity. Overall, once-weekly (1/w) and twice-weekly (2/w) administration of TPTD similarly showed minimal or mild effects on cortical void formation and the osteocytic lacunar-canalicular system, which suggests that there is a dividing point between frequent and infrequent administration of TPTD on cortical bone metabolism. Further investigation is required to uncover the physiological and pharmacological effects of PTH on osteocytes.

## Methods

### Animals and the preparation of bone specimens

Forty female New Zealand white rabbits (Kbl:NZW) were purchased from Kitayama Labs (Nagano, Japan) and allowed to acclimatize with free access to water and food (LRC4, Oriental Yeast Co. ltd., Tokyo, Japan) for three weeks before use. Throughout the experimental study, the animals were housed individually in aluminum cages (81 W × 50D × 35H cm) under a 12-h light/dark cycle with free access to water, but their food intake was restricted to 120 g/day (LRC4, standard diet for rabbits; Oriental Yeast, Tokyo, Japan). Both Animal care staff and those who administer treatments monitored animals twice daily. Health was monitored by weight (once weekly), food intake, and general assessment of animal activity. Animals with a body weight of 3.4–4.6 kg at 6 months of age were divided into four groups by block randomization using SAS, Version 8.2 (SAS Institute Inc., Cary, NC, USA) with tibial BMD and body weight and were subcutaneously injected with 140 μg/kg of TPTD (Asahi Kasei Pharma Corporation, Tokyo, Japan) once-weekly (1/w group, n = 10), 70 μg/kg of TPTD twice-weekly (2/w group, n = 10), and 20 μg/kg of TPTD once-daily (7/w group, n = 10) for four weeks (Fig. 1). In the 1/w and 2/w groups, saline was administered daily, except on the days on which TPTD was administered. Saline injections were administered to control animals (control group, n = 10) once-daily. Based on previous studies, 10 animals in each group were required for bone morphometric evaluation. Calcein (Dojindo Laboratories, Kumamoto, Japan) was subcutaneously injected into each animal at a dose of 10 mg/kg of body weight on days 29 and 4 before sacrifice for bone double labeling (1-25-1-3). It was planned to exclude from the study any individual who experienced severe weight loss or other poor condition from which recovery was not expected during the treatment period, but no such cases occurred.

After the dosing period, the animals were euthanized by exsanguination under anesthesia using thiopental. The left and right tibiae were collected for DXA, three-point bending test, and histomorphometry. The left tibiae were packed in plastic bags and stored at − 30 °C until use in DXA and mechanical testing. The right tibiae were fixed in 70% ethanol, stained with Villanueva bone stain, dehydrated in a graded ethanol series, defatted in acetone, and embedded in polymethyl methacrylate (Wako Pure Chemical Industries, Osaka, Japan). Thin ground sections of 10–20-μm thickness were prepared using a micro-cutting machine and a grinding machine (EXAKT, Germany) from a cross-section at a plane that was 3 mm proximal to the tibiofibular junction and were subjected to bone histomorphometry.

The experimental protocols were approved by the experimental animal ethics committee at the Asahi Kasei Pharma Corporation and were conducted in accordance with the guidelines for the management and handling of experimental animals. Animals were housed under non-specific pathogen-free conditions at Ina Research Inc., accredited by AAALAC (The Association for Assessment and Accreditation of Laboratory Animal Care International). Animal care staff and those who administer treatments were not involved in sample measurements. All sample measurements were performed by objective methods. The reporting in the manuscript follows the recommendations in the ARRIVE guidelines (https://arriveguidelines.org).

### Measurement of BMD

BMDs of the collected left tibiae were monitored using DXA (DCS-600EX-IIIR; Aloka, Tokyo, Japan). Whole samples were scanned with a pitch of 2 mm. BMD (mg/cm^2^) was then calculated from the bone mineral content (mg) and bone area (cm^2^).

### Bone mechanical properties

The fibulae were cut off from the left tibiae using a micromotor (Volvere GX NE22, Nakanishi, Japan). After pre-processing, the samples were subjected to a three-point bending test. The samples were set on supports 32 mm apart and attached to a testing machine (AUTOGRAPH AGS-5kNX, Shimadzu, Japan) such that the dorsal aspects were upward, and load was applied at points 3 mm proximal to the tibiofibular junctions at a constant speed of 10 mm/min. The load and displacement curves were recorded, and the following parameters were calculated using TRAPEZIUM LITE X software (Shimadzu): maximum load (N), stiffness (N/mm), and energy absorption (Energy, mJ). Energy absorption was defined as the energy absorbed until the load reached its recorded maximum value.

### Blood and urine sampling

Blood samples were collected from auricular veins before the initial administration of TPTD on day 1, as well as on days 4, 8, 15, 22, 25, and 29 after the initial administration of TPTD. The samples were centrifuged at 1700×*g* at 4 °C for 15 min, and serum samples were collected to measure OC concentration.

In all groups, the animals were placed in metabolic cages, and urine samples were collected for 24 h before the initial administration of TPTD on day 1, as well as before dosing on days 8, 15, 22, and 29. Urine samples were centrifuged at 400×*g* at 4 °C for 5 min, and the supernatant was collected to measure the concentration of DPD.

### Measurement of markers of bone metabolism

The serum levels of OC, a bone formation marker, were measured using a Gla-osteocalcin ELISA system (Takara Bio, Tokyo, Japan). The urinary level of DPD was measured using Osteolinks DPD (DS Pharma Biomedical) and normalized to creatinine concentration. The assays were performed according to the manufacturer’s instructions.

### Wide-field fluorescence and DIC imaging

Using a wide-field microscopy system, ECLIPSE Ni (Nikon, Tokyo, Japan) equipped with a DIC microscope and objectives (Nikon), Plan Apo λ × 10 [numerical aperture (NA) = 0.45], Plan Apo λ × 20 (NA = 0.75), and Plan Apo λ × 40 (NA = 0.95), fluorescence and DIC imaging were obtained. Filter sets of fluorescein isothiocyanate [excitation: 460–500 nm, dichroic mirror (DM): 505 nm, emission: 510–560 nm; Nikon] and TxRed (excitation: 540–580 nm, DM: 595 nm, emission: 600–660 nm; Nikon) were used for calcein and autofluorescence derived from soft tissue, respectively. Tiling fluorescence and DIC imaging were sequentially performed to acquire the entire, high-contrast view of the tissue sections using the Plan Apo λ × 10 objective (NA = 0.45). The frame size of a single scan was 1280 × 1024 pixels, with an 8-bit color depth and a pixel size of 0.64 μm. Image processing was performed using NIS-Elements AR imaging software (Nikon, Tokyo, Japan).

### Confocal fluorescence imaging

Confocal imaging of Villanueva-stained bone sections was performed using a Nikon confocal laser microscopy system (A1-ECLIPSE Ti2; Nikon). Two objectives were used: Apo 60 × oil DIC N2 (NA = 1.40) and HP Apo TIRF 100 × oil DIC N2 (NA = 1.49). Two laser lines at 488 nm and 561 nm for excitation and two filter cubes at 480 nm/560 nm and 560 nm/640 nm for detection were used. The images with 0.29 μm/pixel for the 60 × objective, 0.10 μm/pixel for the 100 × objective, and a 12-bit color depth were acquired. For, three-dimensional fluorescence morphometry, confocal images were taken with 1.0 and 0.3 μm step sizes for voxel sampling with 60 × and 100 × objectives, respectively.

### Semi-quantitative fluorescence morphometry and automatic morphometric recognition analyses using AI and deep learning

Quantitative topological analyses of fluorescence signals in undecalcified bone sections were performed using commercially available imaging analysis tools (NIS-Elements AR and NIS. ai, Nikon, Tokyo, Japan). The following parameters of the recognized objects were semi-automatically measured: object number, object area, length, outer perimeter, circularity, and smoothness. Examples of the measurement parameters for the analyses of cortical porosity and calcein labeling are schematically illustrated in Supplementary Fig. S1. The binarization of objects was conducted interactively using the fluorescence intensity threshold, object size, and manual histomorphological definition. The recognition of objects was set as follows: cortical bone porosity, threshold 12, size > 90.0 µm; calcein labeling, threshold 200, size > 15.0 µm. Automatic morphometric recognition of osteocytes and the Haversian canal was performed using AI and deep learning methods (NIS.ai, segment ai). As shown in the flowchart in Fig. 7a, we first created AI training data to properly recognize signals derived from osteocytic lacunae and Haversian canals after conducting morphological recognition learning more than 1000 times. As shown in Fig. 8, 16 or more training images showed significantly high and satisfying object recognition rates; therefore, we created AI training datasets with 16 images and used them for our analyses. Using this established AI-training data, we binarized osteocytic lacunae and Haversian canals properly and automatically and conducted quantitative morphometric analyses. Using this AI-driven measurement, the following parameters of the recognized objects were measured and statistically compared: object number, object area, length, width, perimeter, and circularity. Examples of two-dimensional and three-dimensional measurement parameters for the analyses of osteocytic lacunae are schematically illustrated in Figs. 7c, 12b,c. In the measurement of two-dimensional and three-dimensional osteocytic lacunae, the regions of interest were rectangular (vertical 0.1 mm; horizontal 0.2 mm) and square (vertical 0.1 mm; horizontal 0.1 mm) forms, respectively. The region of interest of the Haversian canal was a square form (vertical 0.3 mm; horizontal 0.3 mm).

### Three-dimensional reconstruction and morphometry of the osteocytic lacunar-canalicular system

The three-dimensional fluorescence images acquired with HP Apo TIRF 100 × oil DIC N2 (NA = 1.49) were constructed from z-series images using the IMARIS software program (Bitplane, Zurich, Switzerland) as described previously^52^. To separate the lacunae and canaliculi, start and end points were set at 0.6 and 0.3 µm, respectively.

### Statistical analysis

All data are presented as the mean and standard error (SE) (n = 10). The effects of TPTD on bone specimens were evaluated using analysis of variance. Dunnett’s test was used to compare the treatment and vehicle control groups (Tibial BMDs, mechanical properties and bone metabolic markers: SAS, Version 9.4, the others: Medical and Pharmaceutical Statistics). Statistical significance was set at p < 0.05.

## Supplementary Information

Supplementary Figures.

## Data Availability

The data that support the findings of this study are available from the corresponding authors, A.T., R.T-K., T.I. upon reasonable request.
