# Supplementary material for: Expansion of the osteocytic lacunar-canalicular system involved in pharmacological action of PTH revealed by AI-driven fluorescence morphometry in female rabbits
Source: Sci Rep. 2022 Oct 7;12:16799. doi: 10.1038/s41598-022-20793-5 (PMC9546928; doi:10.1038/s41598-022-20793-5)
Supplement: Supplementary file 1 — Supplementary Figures. [file 41598_2022_20793_MOESM1_ESM.pptx]

## Slide 1
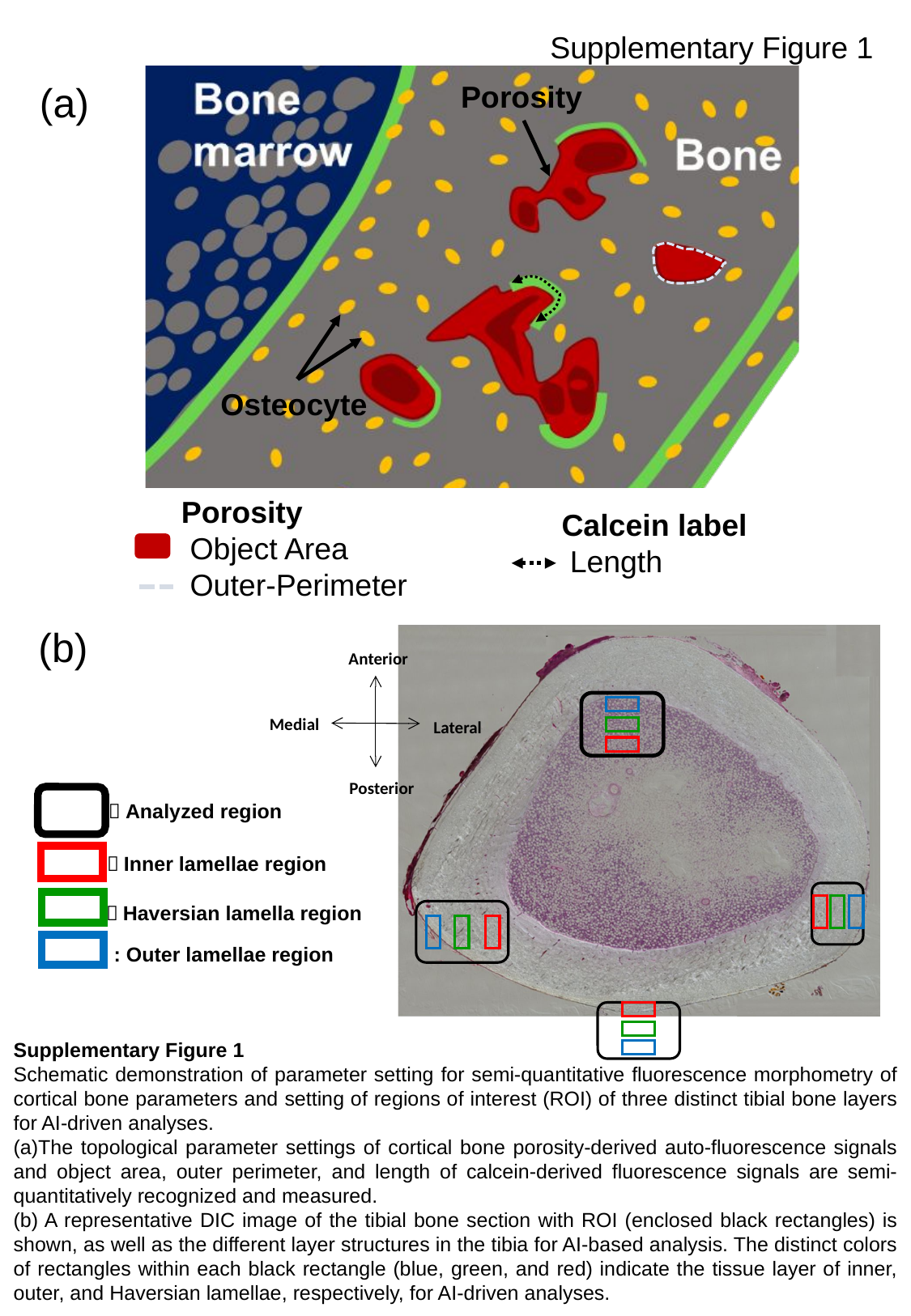

Supplementary Figure 1
(a)
Porosity
Osteocyte
Porosity
 Object Area
 Outer-Perimeter
Calcein label
 Length
(b)
Anterior
Medial
Lateral
Posterior
：Analyzed region
：Inner lamellae region
：Haversian lamella region
: Outer lamellae region
Supplementary Figure 1
Schematic demonstration of parameter setting for semi-quantitative fluorescence morphometry of cortical bone parameters and setting of regions of interest (ROI) of three distinct tibial bone layers for AI-driven analyses.
(a)The topological parameter settings of cortical bone porosity-derived auto-fluorescence signals and object area, outer perimeter, and length of calcein-derived fluorescence signals are semi-quantitatively recognized and measured.
(b) A representative DIC image of the tibial bone section with ROI (enclosed black rectangles) is shown, as well as the different layer structures in the tibia for AI-based analysis. The distinct colors of rectangles within each black rectangle (blue, green, and red) indicate the tissue layer of inner, outer, and Haversian lamellae, respectively, for AI-driven analyses.

## Slide 2
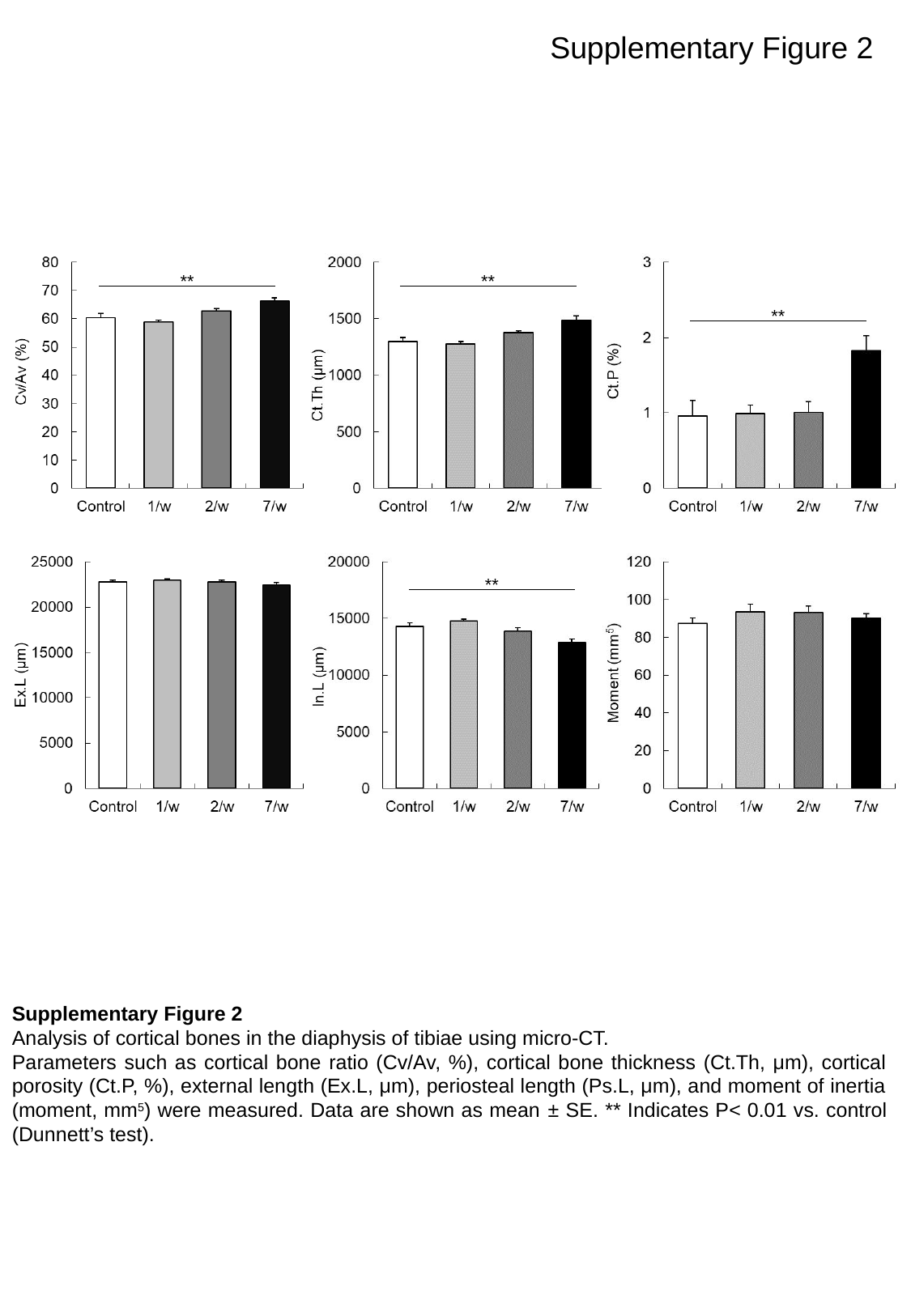

Supplementary Figure 2
**
**
**
**
Supplementary Figure 2
Analysis of cortical bones in the diaphysis of tibiae using micro-CT.
Parameters such as cortical bone ratio (Cv/Av, %), cortical bone thickness (Ct.Th, μm), cortical porosity (Ct.P, %), external length (Ex.L, μm), periosteal length (Ps.L, μm), and moment of inertia (moment, mm5) were measured. Data are shown as mean ± SE. ** Indicates P< 0.01 vs. control (Dunnett’s test).

## Slide 3
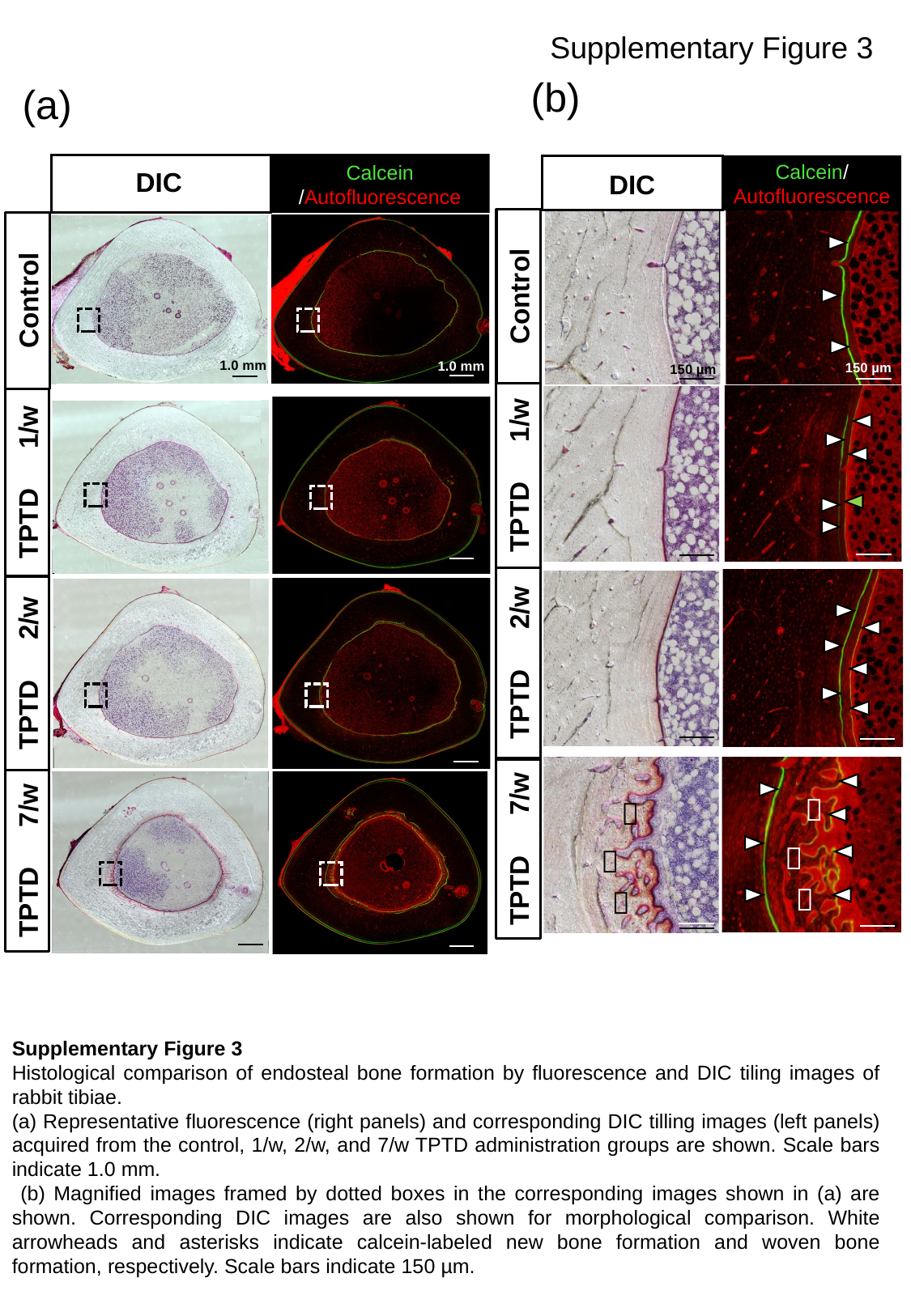

Supplementary Figure 3
(b)
(a)
Calcein
/Autofluorescence
Calcein/
Autofluorescence
DIC
DIC
Control
Control
1.0 mm
150 µm
1.0 mm
150 µm
TPTD　1/w
TPTD　1/w
TPTD　2/w
TPTD　2/w
＊
＊
TPTD　7/w
＊
＊
TPTD　7/w
＊
＊
Supplementary Figure 3
Histological comparison of endosteal bone formation by fluorescence and DIC tiling images of rabbit tibiae.
(a) Representative fluorescence (right panels) and corresponding DIC tilling images (left panels) acquired from the control, 1/w, 2/w, and 7/w TPTD administration groups are shown. Scale bars indicate 1.0 mm.
 (b) Magnified images framed by dotted boxes in the corresponding images shown in (a) are shown. Corresponding DIC images are also shown for morphological comparison. White arrowheads and asterisks indicate calcein-labeled new bone formation and woven bone formation, respectively. Scale bars indicate 150 µm.

## Slide 4
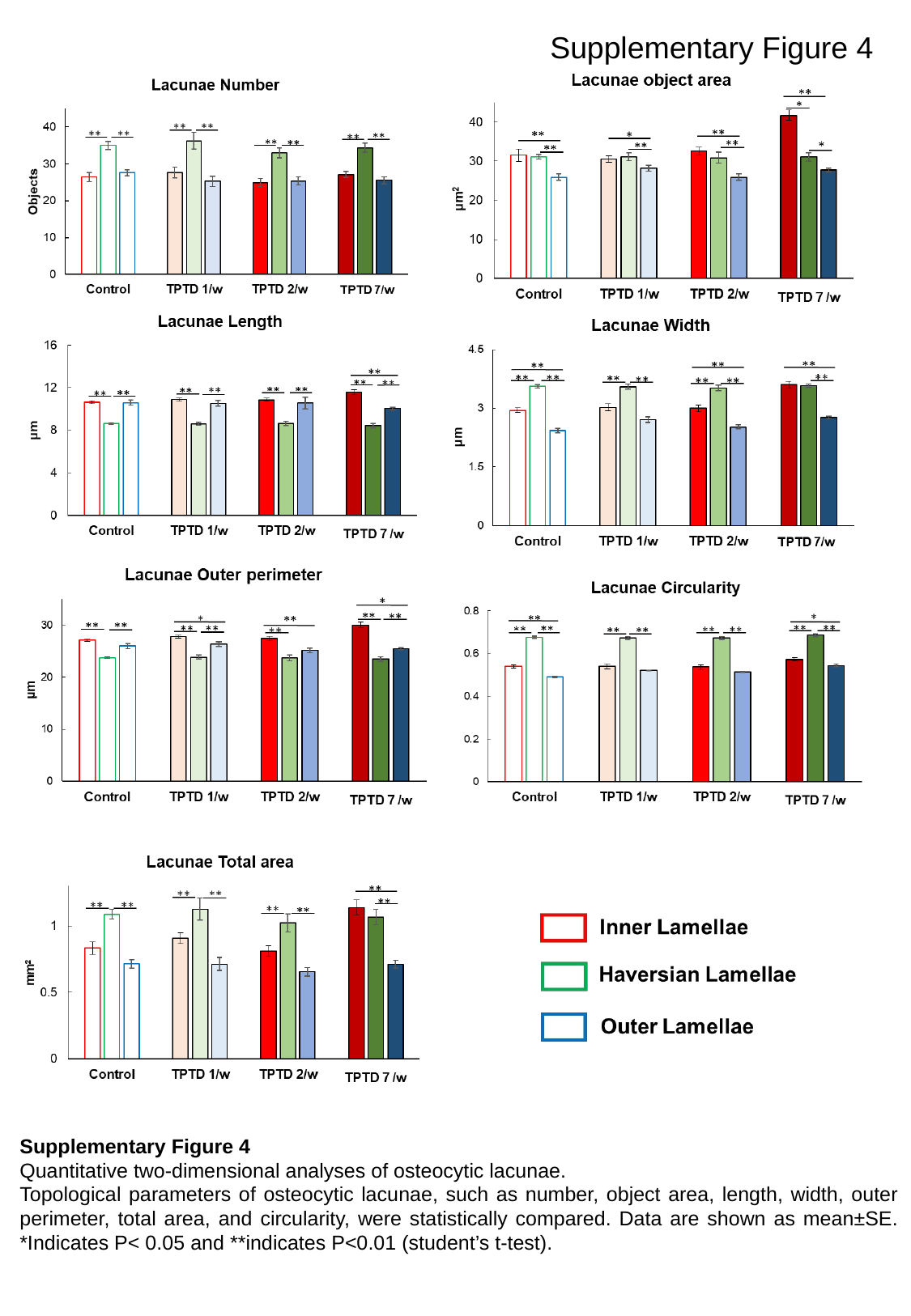

Supplementary Figure 4
Supplementary Figure 4
Quantitative two-dimensional analyses of osteocytic lacunae.
Topological parameters of osteocytic lacunae, such as number, object area, length, width, outer perimeter, total area, and circularity, were statistically compared. Data are shown as mean±SE. *Indicates P< 0.05 and **indicates P<0.01 (student’s t-test).

## Slide 5
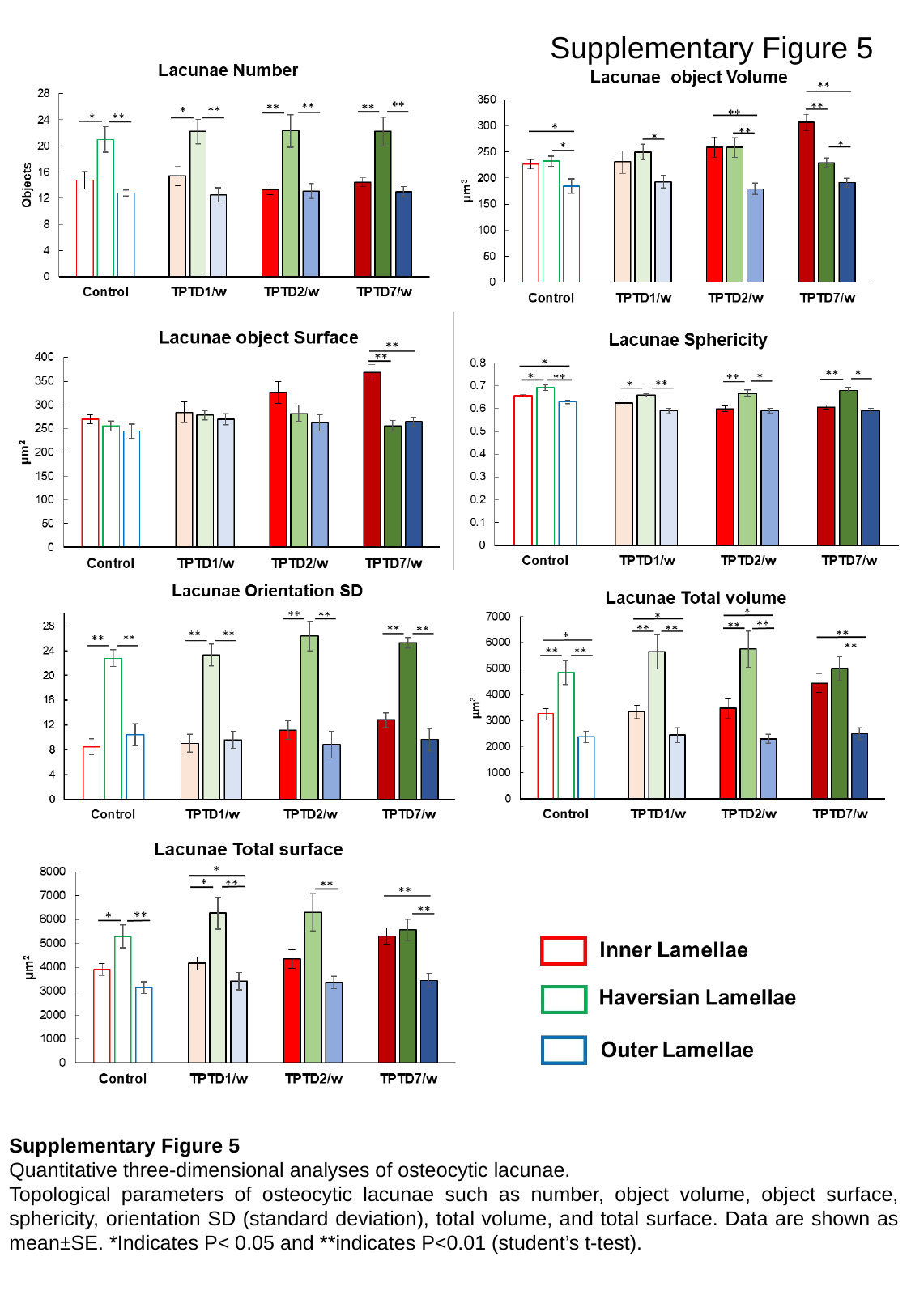

Supplementary Figure 5
Supplementary Figure 5
Quantitative three-dimensional analyses of osteocytic lacunae.
Topological parameters of osteocytic lacunae such as number, object volume, object surface, sphericity, orientation SD (standard deviation), total volume, and total surface. Data are shown as mean±SE. *Indicates P< 0.05 and **indicates P<0.01 (student’s t-test).

## Slide 6
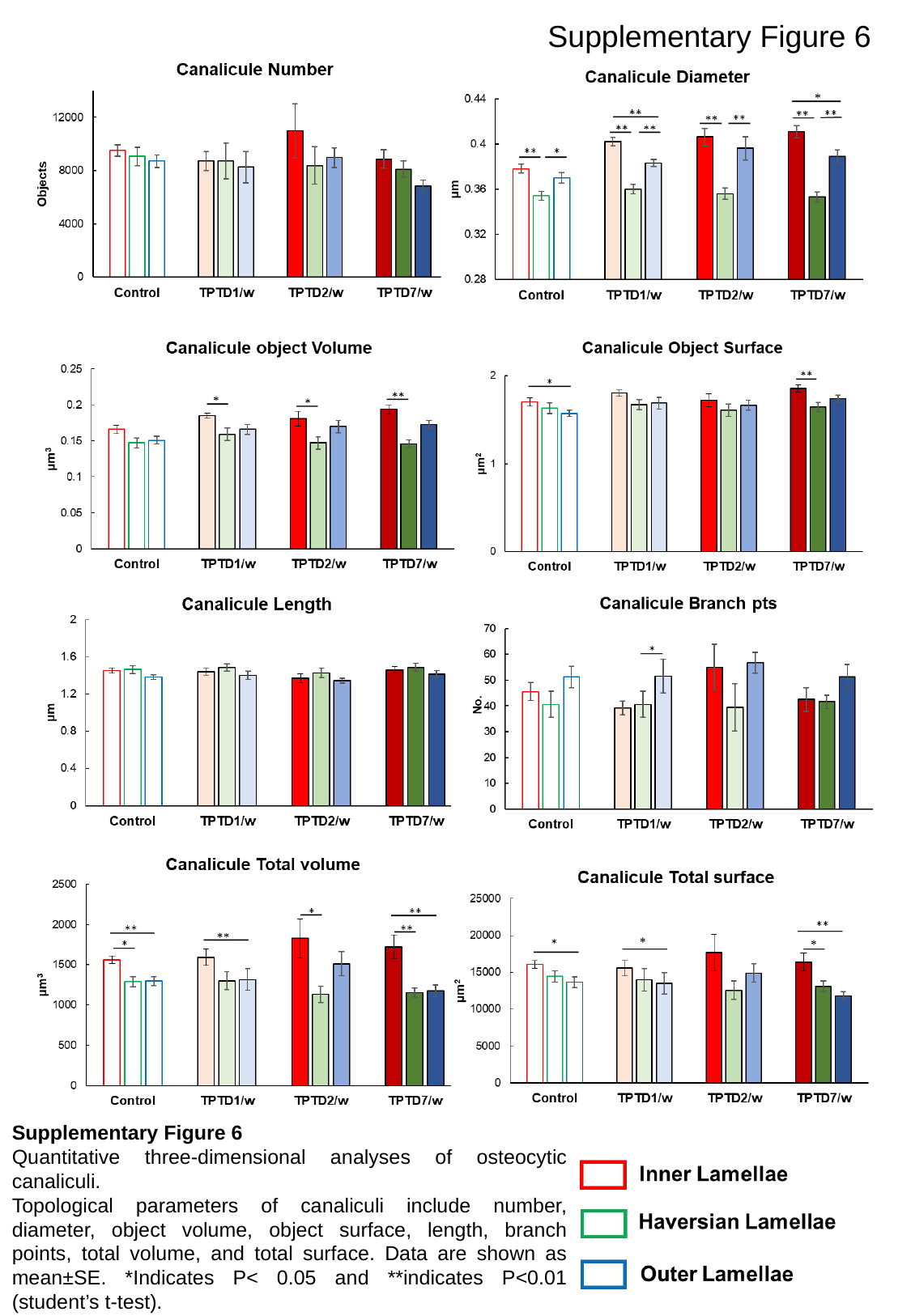

Supplementary Figure 6
Supplementary Figure 6
Quantitative three-dimensional analyses of osteocytic canaliculi.
Topological parameters of canaliculi include number, diameter, object volume, object surface, length, branch points, total volume, and total surface. Data are shown as mean±SE. *Indicates P< 0.05 and **indicates P<0.01 (student’s t-test).
